# The Utility of a Point-of-Care Transcranial Doppler Ultrasound Management Algorithm on Outcomes in Pediatric Asphyxial Out-of-Hospital Cardiac Arrest – An Exploratory Investigation

**DOI:** 10.3389/fmed.2021.690405

**Published:** 2022-01-28

**Authors:** Jainn-Jim Lin, Hsuan-Chang Kuo, Shao-Hsuan Hsia, Ying-Jui Lin, Huei-Shyong Wang, Mei-Hsin Hsu, Ming-Chou Chiang, Oi-Wa Chan, En-Pei Lee, Kuang-Lin Lin

**Affiliations:** ^1^Division of Pediatric Critical Care and Pediatric Neurocritical Care Center, Chang Gung Children's Hospital and Chang Gung Memorial Hospital, Chang Gung University College of Medicine, Taoyuan, Taiwan; ^2^Graduate Institute of Clinical Medical Sciences, Chang Gung University, College of Medicine, Taoyuan, Taiwan; ^3^Division of Pediatric Neurology, Chang Gung Children's Hospital and Chang Gung Memorial Hospital, Chang Gung University College of Medicine, Taoyuan, Taiwan; ^4^Department of Respiratory Therapy, Chang Gung Children's Hospital and Chang Gung Memorial Hospital, Chang Gung University College of Medicine, Taoyuan, Taiwan; ^5^Division of Cardiology, Department of Pediatrics, Kaohsiung Chang Gung Memorial Hospital and Chang Gung University College of Medicine, Kaohsiung, Taiwan; ^6^Division of Critical Care, Department of Pediatrics, Kaohsiung Chang Gung Memorial Hospital and Chang Gung University College of Medicine, Kaohsiung, Taiwan; ^7^Division of Neurology, Department of Pediatrics, Kaohsiung Chang Gung Memorial Hospital and Chang Gung University College of Medicine, Kaohsiung, Taiwan; ^8^Division of Neonatology, Chang Gung Children's Hospital and Chang Gung Memorial Hospital, Chang Gung University College of Medicine, Taoyuan, Taiwan; ^9^Study Group for Intensive and Integrated Care of Pediatric Central Nervous System, Chang Gung Children's Hospital, Taoyuan, Taiwan

**Keywords:** point-of-care, transcranial Doppler ultrasound, asphyxial, pediatric, out-of-hospital cardiac arrest

## Abstract

**Background:**

Transcranial Doppler ultrasound is a sensitive, real time tool used for monitoring cerebral blood flow; it could provide additional information for cerebral perfusion in cerebral resuscitation during post cardiac arrest care. The aim of the current study was to evaluate the utility of a point-of-care transcranial Doppler ultrasound management algorithm on outcomes in pediatric asphyxial out-of-hospital cardiac arrest.

**Methods:**

This retrospective cohort study was conducted in two tertiary pediatric intensive care units between January 2013 and June 2018. All children between 1 month and 18 years of age with asphyxial out-of-hospital cardiac arrest and a history of at least 3 min of chest compressions, who were treated with therapeutic hypothermia and survived for 12 h or more after the return of circulation were eligible for inclusion.

**Results:**

Twenty-one patients met the eligibility criteria for the study. Sixteen (76.2%) of the 21 children were male, and the mean age was 2.8 ± 4.1 years. Seven (33.3%) of the children had underlying disorders. The overall 1-month survival rate was 52.4%. Twelve (57.1%) of the children received point-of-care transcranial Doppler ultrasound. The 1-month survival rate was significantly higher (*p* = 0.03) in the point-of-care transcranial Doppler ultrasound group (9/12, 75%) than in the non-point-of-care transcranial Doppler ultrasound group (2/9, 22.2%).

**Conclusions:**

Point-of-care transcranial Doppler ultrasound group was associated with a significantly better 1-month survival rate compared with no point-of-care transcranial Doppler ultrasound group in pediatric asphyxial out-of-hospital cardiac arrest.

## Background

Asphyxia is the most common cause of pediatric out-of-hospital cardiac arrest (OHCA). Despite advances in resuscitation, patients who survive pediatric OHCA often suffer from high mortality and severe neurological sequelae (1–3). The neurological prognosis after pediatric OHCA depends on the duration and severity of global brain ischemia and hypoxia (4, 5). Effective cerebral perfusion after resuscitation has a notable influence on neurological outcomes (6–8). However, the return of spontaneous circulation (ROSC) does not automatically restore adequate cerebral perfusion. Actual cerebral blood flow is difficult to measure in children with critical illness because accurate measurement methods, including single-photon emission computed tomography, positron emission tomography, and radionuclide angiography are complex techniques, which are not suitable for routine use in clinical practice. The identification of a more immediate, non-invasive, bedside approach to complement these existing methods is therefore of importance.

Transcranial Doppler ultrasonography (TCD) provides an easily applicable, bedside technique for the evaluation and monitoring of cerebral blood flow in the main cerebral arteries. Point-of-care TCD has many useful applications in the day-to-day bedside assessment of cerebral hemodynamic status in neurocritical care practice, including post cardiac arrest care (9–14). A patient's cerebral hemodynamic status after resuscitation can be determined using the pattern of Doppler spectral waveform, the pulsatility index (PI) and the mean flow velocity of the main cerebral arteries following TCD examination (9–12). Using serial TCD examinations, changes in the Doppler spectral waveform, PI and mean flow velocity strongly reflect hemodynamic fluctuations during post-cardiac arrest care (10, 11, 13–16).

Therefore, point-of-care TCD might guide cerebral resuscitation to improve the patient's cerebral hemodynamic status during post cardiac arrest care. However, only a few studies have reported on point-of-care TCD guided cerebral resuscitation in pediatric asphyxial OHCA survivors (14). A consensus stepwise management algorithm was designed, based on the experiences described in the authors previously published article, for the intensive care of unconscious pediatric asphyxial OHCA cases (10). This was available for use in the author's pediatric intensive care unit since 2013. The aim of the present exploratory analysis was to evaluate the effect of point-of-care TCD guided cerebral resuscitation on patient outcomes in pediatric asphyxial OHCA survivors.

## Methods

### Patient Population

**Figure 1:**
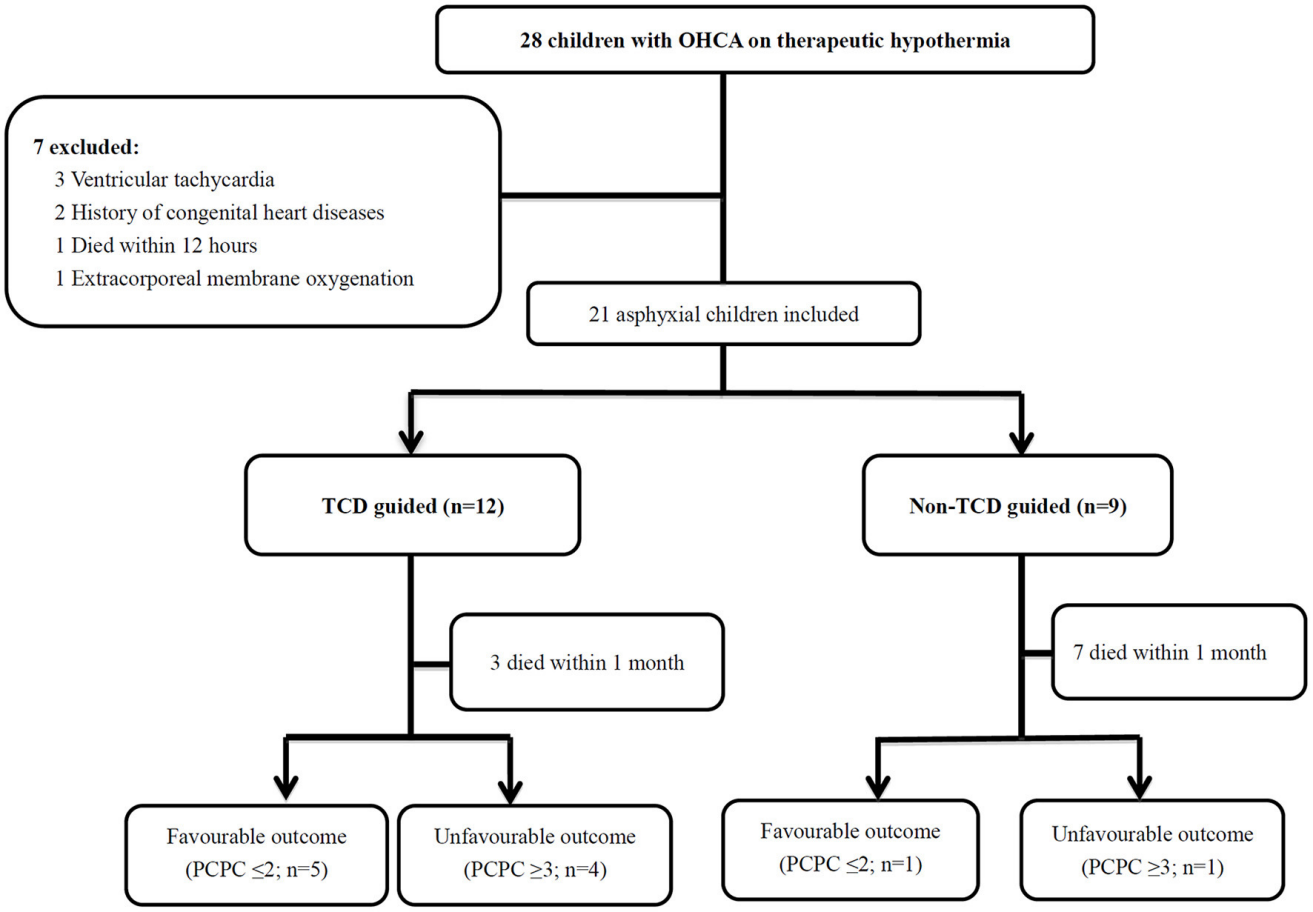
Twenty-eight patients with OHCA with therapeutic hypothermia were identified. A total of 7 children were excluded, and 21 asphyxial OHCA comatose patients were enrolled, including 12 patients who received the TCD guided cerebral resuscitation protocol and 9 patients without the TCD guided cerebral resuscitation protocol. The patients with 6-month neurological outcomes included those who died during the follow-up period. The survival rate was significantly higher (*p* = 0.03) in the TCD guided group (9/12, 75%) compared with the non-TCD guided group (2/9, 22.2%). OHCA, out-of-hospital cardiac arrest; PCPC, pediatric cerebral performance category; TCD, Transcranial Doppler ultrasound.

The present investigation was a retrospective cohort study using chart reviews of asphyxial OHCA at two pediatric intensive care units of Chang Gung Children's Hospital (Linkou and Kaohsiung branches), between January 1st 2013 and June 30th 2018 (Figure 1). OHCA was defined as patients who had successfully been resuscitated with ROSC following cardiac arrest, and for whom chest compressions were initiated before arriving at the hospital (1, 2, 10). Asphyxial cardiac arrest was defined as resuscitation secondary to preceding acute respiratory failure.

Patients were eligible for the study if they met the following criteria: (1) aged between 1 month and 18 years; (2) duration of cardiac arrest was at least 3 min and they survived for 12 h or more after the ROSC; (3) comatose status (Glasgow coma scale [GCS] score) ≤8 after ROSC; (4) receiving therapeutic hypothermia (1, 2, 10). Patients were excluded if they met any of the following criteria: (1) were older than 18 years; (2) had hemodynamic instability refractory to intensive care and died within 12 h; (3) were not in a coma after resuscitation (GCS >8); (4) were known to have pre-existing degenerative neurological diseases; and (5) had ventricular fibrillation, a history of congenital heart disease or were on extracorporeal membrane oxygenation. If a patient experienced more than one resuscitation during the study period, only the first resuscitation meeting the eligibility criteria was included. The present study was approved by the Chang Gung Memorial Hospital Institutional Review Board (IRB numbers: 201700975B0, 201700976B0, 201700977B0 and 201900302B0).

### Post-cardiac Arrest Care

The two study institutions follow general principles of post cardiac arrest care after resuscitation (2, 10). In general, systolic and mean arterial blood pressure are maintained at or above the lower limit in children. All patients were intubated with mechanical ventilation to maintain an arterial oxygen saturation of ≥94% and normocarbia (PaCO_2_ 35–40 mmHg). The hemoglobin level was also maintained above 10 gdL^−1^ (1, 2, 10). With regard to the treatment of increased ICP, the head of the bed was routinely elevated to >30°. Hyperosmolar therapy with mannitol and/or 3% hypertonic saline was used to lower ICP. The goal osmolality was between 300 and 320 mosm/KgH_2_O and the goal serum sodium was between 145 and 155 mEq/L (17, 18). All patients also received therapeutic hypothermia, and were sedated with midazolam and/or received neuromuscular blockers.

### Point-of-Care TCD Guided Cerebral Resuscitation Protocol

**Figure 2:**
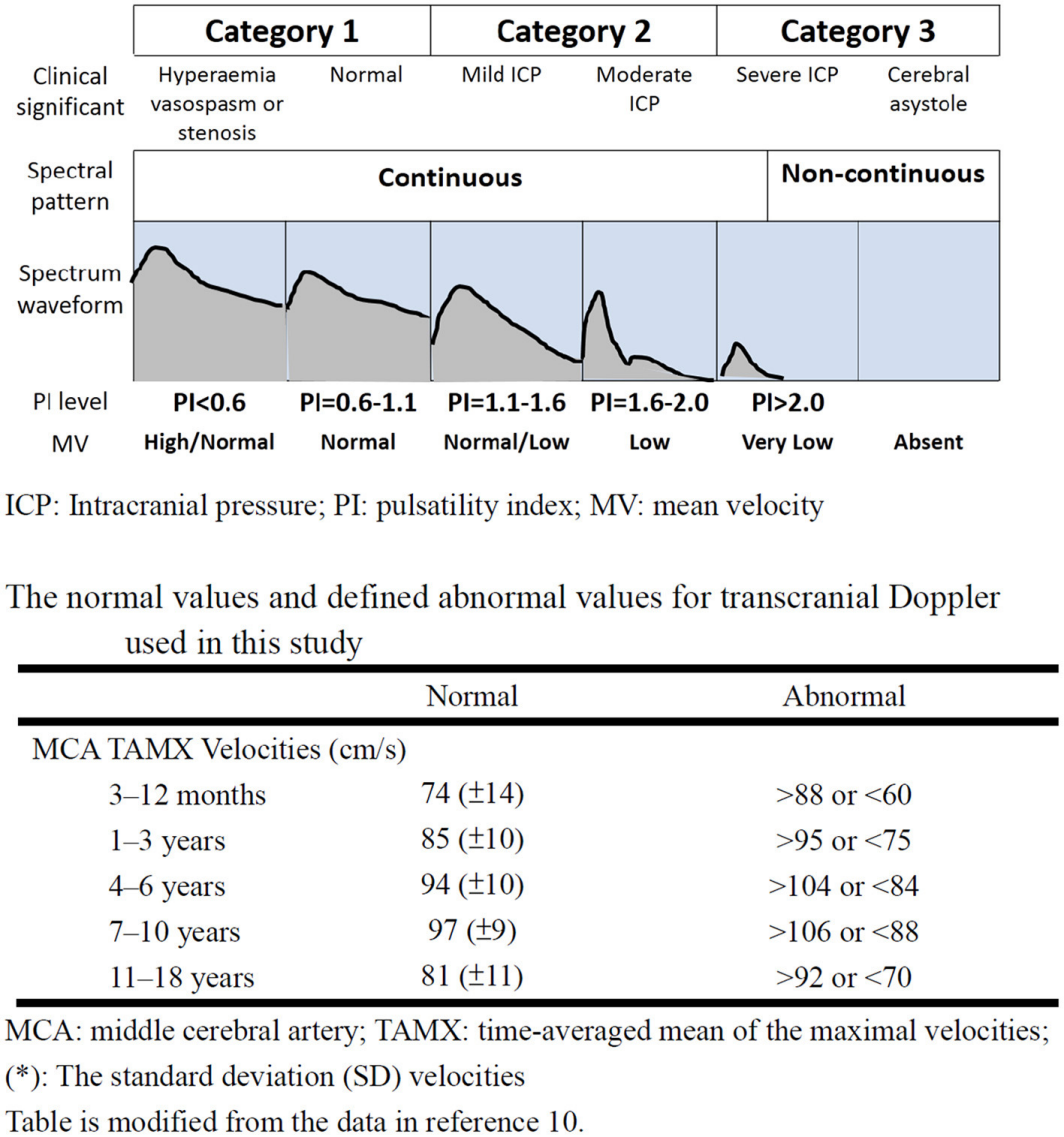
The category of transcranial Doppler ultrasound findings according to spectral pattern, PI and mean flow velocities after the ROSC. The figure was modified from references 9 and 11. ICP, Intracranial pressure; PI, pulsatility index; MV, mean velocity; ROSC, return of spontaneous circulation.

The point-of-care TCD guided cerebral resuscitation protocol was performed at the Linkou branch of the hospital. Serial TCD examinations of the bilateral middle cerebral arteries (MCAs) were performed using a 2 MHz probe (128XP; Acuson, Mountain View, CA, USA) or an S5-1 MHz probe (Philips, Andover, MA, USA). Point-of-care TCD was performed in three phases of therapeutic hypothermia: a pre-hypothermia phase, hypothermia phase (72 h when the body temperature reached 33°C), and a rewarming phase. TCD findings were provided to the clinical teams at least once every day, or more often if any changes occurred. For each TCD investigation, the pattern of Doppler spectral waveform, values of the peak, end diastolic and mean flow velocity were recorded and the PI was calculated (9–12). The pattern of Doppler spectral waveform was divided into discontinuous and continuous patterns. A discontinuous pattern was defined as a pattern with the loss of diastolic flow, the appearance of retrograde diastolic flow, or no detectable flow. The normal values and defined abnormal values for PI and mean flow velocity in the MCA used in the current study are summarized in Figure 2 (10). The TCD findings were scored as the most severe findings in different phases. If the PI and mean flow velocity were different in bilateral MCA, the most severely abnormal values were used as the severity of this phase.

According to TCD data and the prognosis in previous literature, the TCD finding was divided into three categories: category (1) continuous waveform with PI <1.1 and normal or high mean flow velocity, category (2) continuous waveform with PI between 1.1 and 2.0 with low or normal mean flow velocity, category (3) discontinuous waveform or continuous waveform with PI >2.0 and low mean flow velocity (Figure 2) (10).

**Figure 3:**
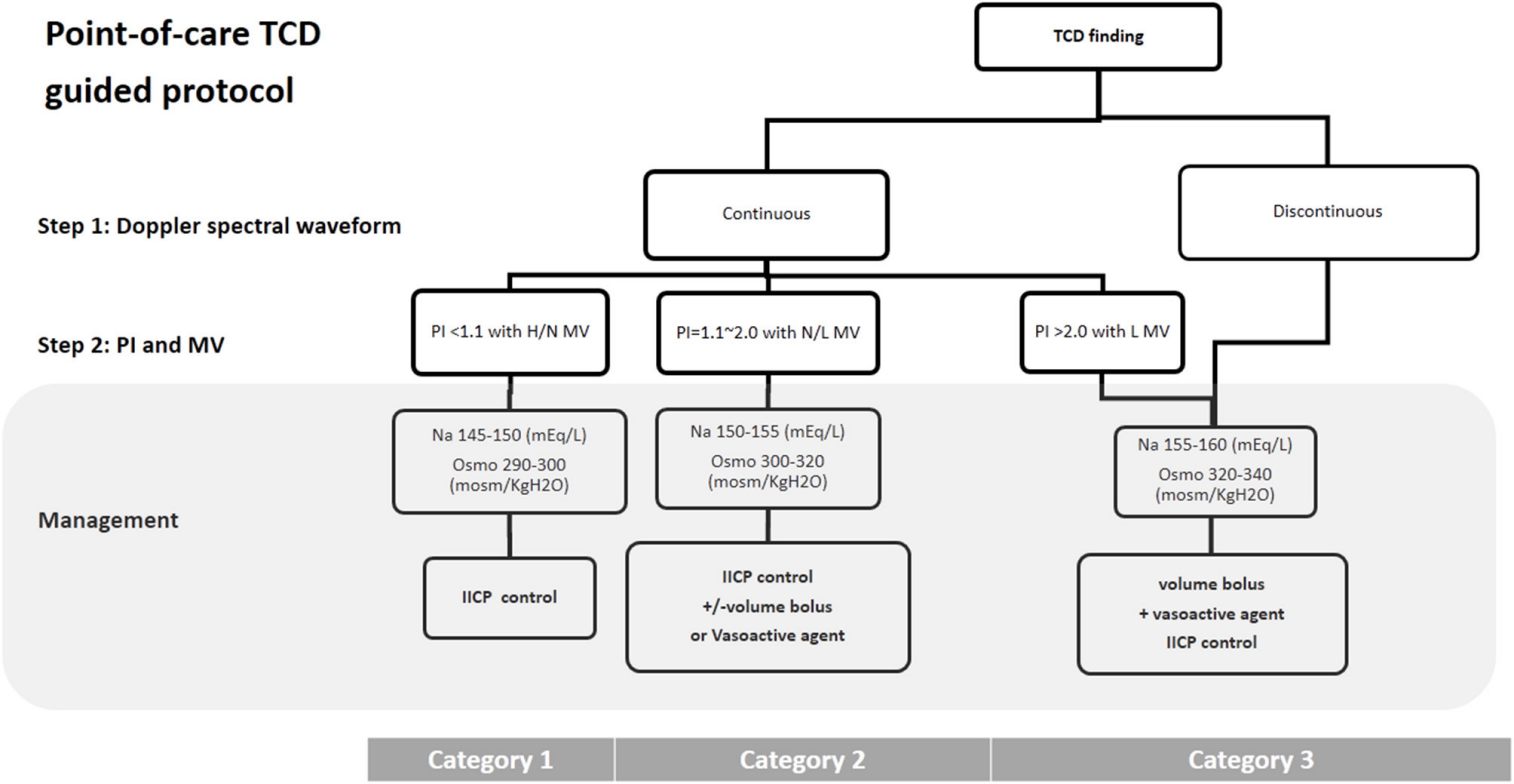
TCD guided cerebral resuscitation protocol. A stepwise management algorithm shows the clinical treatment strategy according to the pattern of Doppler spectral waveform, PI and MV of middle cerebral artery on TCD examination. H, high; N, Normal; L, low; IICP, increased intracranial pressure; TCD, Transcranial Doppler ultrasound; PI, pulsatility index; MV, mean velocity; Osmo, osmolality.

Hypertonic saline sliding scale protocol was used to achieve target sodium levels at 3 different categories, which was modified from previously published stepwise protocol for intracranial pressure control in head-injured pediatric patients (19, 20). The goal of hyperosmolar therapy for category 1 was maintenance of serum osmolality between 290 and 300 mOsm/kg H_2_O and serum sodium between 145 and 150 mEq/L, and systolic and mean arterial blood pressures at or above the lower limit in children (19, 20). For category 2, the goal of hyperosmolar therapy was maintenance of serum osmolality between 300 and 320 mOsm/kg H_2_O and serum sodium between 150 and 155 mEq/L (19, 20), and achieving the lower limit of mean flow velocity on TCD by increasing vasoactive medications or administering fluids and blood products (19–21). For category 3, vasoactive medications combined with fluids or blood products were administered to achieve higher systolic and mean arterial blood pressures (8, 19–21). The goal of the intensive care guided by TCD findings were: (1) to achieve a continuous waveform and at least the lower limit of mean flow velocity; (2) to maintain the serum osmolality at 320–340 mOsm/kg H_2_O and serum sodium at 155–160 mEq/L (19, 20) (Figure 3).

### Data Collection

The following information was collected for all patients: (1) demographics and pre-existing diseases; (2) event characteristics, such as first-monitored rhythm during arrest and the duration of cardiopulmonary resuscitation; (3) variables after resuscitation. The primary outcome was the survival rate at 30 days following cardiac arrest.

The primary cause of death within 1 month was categorized into 3 groups: (1) brain death and withdrawal for poor neurological prognosis, (2) cardiovascular failure/futility, and (3) and respiratory failure/futility (2). The secondary outcome was duration of hospitalization and neurological outcome, which was assessed using Pediatric Cerebral Performance Category (PCPC) scores according to the recommendations for outcome assessments in comatose cardiac arrest patients in children who survived for 6 months after the events and in those who died during follow-up (1, 2, 10). Neurological outcomes were dichotomized as either a favorable prognosis (PCPC ≤2) or an unfavorable prognosis (PCPC ≥3).

### Statistical Analysis

The patient characteristics in each study group are represented as descriptive statistics, and the data are presented as the mean ± standard deviation (SD). The effects of point-of-care TCD guided cerebral resuscitation on the 1-month survival rate and 6-months neurological outcomes were analyzed. Between group differences were analyzed using the chi-squared test or Fisher's exact test for categorical variables, and the Student's *t*-test for normally distributed continuous variables. The Mann-Whitney U test was used for non-normally distributed data. Associations between the outcomes of patients in the point-of-care TCD guided and no point-of-care TCD guided groups were determined using univariate analysis. Statistical analyses were performed using SPSS statistical software, version 23.0 (IBM, Inc., Chicago, IL). A two-sided *p* < 0.05 was considered to indicate a statistically significant difference.

## Results

### Patient Profile

During the 5.5-year study period, 74 OHCA patients were identified, of whom 28 (37.8%) children were treated with therapeutic hypothermia. Twenty-one (75%) of the 28 children treated with therapeutic hypothermia met the study inclusion criteria. Seven children were excluded, including 3 with ventricular tachycardia, 2 with a history of congenital heart disease, 1 who died within 12 h due to refractory cardiogenic shock despite the use of vasopressor and/or inotropic agents, and 1 who received extracorporeal membrane oxygenation (Figure 1).

Twelve (57.1%) of the 21 patients received point-of-care TCD guided cerebral resuscitation and the remaining 9 (42.9%) received no point-of-care TCD guided cerebral resuscitation. Thirteen (61.9%) of the 21 events were bystander-witnessed cardiac arrest, however only 7 (33.3%) of the 21 bystanders performed cardiopulmonary resuscitation (CPR). The first documented arrest rhythm was described as asystole in 19 (90.5%) patients and bradycardia/pulseless electrical activity in 2 (9.5%) patients. There were no significant differences in the demographic data between the point-of-care TCD guided and no point-of-care TCD guided groups (Table 1).

**Table 1:** Characteristics of the 21 children with asphyxial out-of-hospital cardiac arrest receiving therapeutic hypothermia.

**Characteristic**	**Point-of-care TCD guided** **(***n*** = 12)**	**No point-of-care TCD guided** **(***n*** = 9)**	***p*** **-value**
Gender			1.000
Female	3 (25%)	2 (22.2%)	
Male	9 (75%)	7 (77.8%)	
Age			0.120
1 month-11 months	9 (75%)	5 (55.6%)	
1–4 years	2 (16.7%)	0 (0%)	
5–8 years	1 (8.3%)	1 (11.1%)	
9–18 years	0 (0%)	3 (33.3%)	
Chronic pre-existing illness			0.670
No	8 (66.7%)	6 (66.7%)	
Respiratory	1 (8.3%)	2 (22.2%)	
Neurologic	2 (16.7%)	1 (11.1%)	
Other	1 (8.3%)	0 (0%)	
Bystander-witnessed cardiac arrest	7 (58.3%)	6 (66.7%)	1.000
Bystander performed CPR	3 (25%)	4 (44.4%)	0.397
Initial rhythm			1.000
Asystole	11 (91.7%)	8(88.9%)	
Bradycardia/PEA	1 (8.3%)	1 (11.1%)	
**Characteristics during resuscitation**
Interval of CPR to ROSC (min)	27.33 ± 19.61	17.44 ± 8.66	0.176
Serum pH	7.047 ± 0.217	7.093 ± 0.162	0.617
Initial glucose (mg/dL)	259.25 ± 135.31	239.43 ± 138.74	0.764
Initial lactate (mmol/L)	84.89 ± 46.86	82.56 ± 51.17	0.919
Post-cardiac arrest GCS	3.50 ± 1.16	3.00 ± 0.00	0.166
PRISM III	37.08 ± 7.44	42.33 ± 3.20	0.063
PELODS	38.25 ± 8.83	43.77 ± 4.40	0.078
**Treatments**
VIS^#^	19.87 ± 8.96	25.83 ± 14.25	0.291
Ventilator duration (days)	21.42 ± 19.06	10.67 ± 9.08	0.106
**IICP control (mannitol+3% NaCL)**
Serum sodium (mEq/L)^##^	157.0 ± 6.49	153.55 ± 6.48	0.244
Serum osmolality (mOsm/kg H_2_O)^##^	324.27 ± 17.36	326.20 ± 26.46	0.863
**Outcomes**
Hospital length of stay (days)	32.17 ± 19.82	11.67 ± 9.12	0.006^*^
1-month survival	9 (75%)	2 (22.2%)	0.030^*^
**Cause of 1-month death**
Brain death or withdrawal for poor neurologic prognosis	2 (66.7%)	5 (71.4%)	
Cardiovascular failure/futility	1 (33.3%)	1 (14.3%)	
Respiratory failure/futility	0 (0%)	1 (14.3%)	
**6-month neurological outcomes** **(***n*** = 21)**^a^
Favorable prognosis (PCPC score ≤2)	5 (41.7%)	1 (11.1%)	0.177
Unfavorable prognosis (PCPC score ≥3)	7 (58.3%)	8 (88.9%)	

*TCD, transcranial Doppler ultrasound; CPR, cardiopulmonary resuscitation; PEA, pulseless electrical activity; ROSC, return of spontaneous circulation; GCS, Glasgow Coma Scale; PRISM, pediatric risk of mortality; PELODS, pediatric logistic organ dysfunction scores; TTM, Targeted temperature management; VIS, Vasoactive-Inotropic score; IICP, increased intracranial pressure; PCPC, pediatric cerebral performance category*.

# *The maximum vasoactive-inotropic score level during the first 5 days*.

## *The maximum serum level of sodium and osmolality during the first 5 days*.

a *The patients with 6-month neurological outcomes included those who died during the acute and follow-up period. In the TCD guided group, one patient died during the follow-up period. In the Non-TCD guided group, no patient died during the follow-up period*.

* *p < 0.05: statistically significant*.

### Variables and Treatment During and After Resuscitation

The mean duration of cardiac arrest before the return of ROSC was 23.1 min (range 8–81 min), and there was a longer duration of cardiac arrest in the point-of-care TCD guided group (mean ± SD, 27.33 ± 19.61 min) compared with the no point-of-care TCD guided group (17.44 ± 8.66 min), but this difference was not statistically significant (*p* = 0.176). Serum lactate and glucose levels immediately after resuscitation were similar between the two groups. The baseline patient characteristics were also similar between the two groups, suggesting a similar severity of illness after resuscitation. The event characteristics during resuscitation are listed in Table 1. Detailed clinical data and characteristics observed during resuscitation of the 21 patients with asphyxia OHCA are listed in Additional Table 1.

### Post-cardiac Arrest Care

In terms of treatment, all patients received therapeutic hypothermia and vasoactive and/or inotropic medications. All patients in both groups also received anti-increased intracranial pressure drugs, such as mannitol and/or hypertonic saline. There was a trend of lower maximum vasoactive-inotropic score (19.87 ± 8.96) and a higher serum sodium level (157.0 ± 6.49 mEq/L) during the first 5 days in the point-of-care TCD guided group compared to the no point-of-care TCD guided group (25.83 ± 14.25 and 153.55 ± 6.48 mEq/L), but this difference was not statistically significant (*p* = 0.291 and 0.244, respectively). There were also no significant differences in the additional treatment strategies used in both groups (Table 1).

### Survival Rate and Functional Outcomes

The primary cause of death within 1 month was brain death or withdrawal of life-sustaining therapy owing to a poor neurological prognosis (66.7% in the point-of-care TCD guided group and 71.4% in the no point-of-care TCD guided group) (Table 1). The overall 1-month survival rate was 52.4%. The survival rate was significantly higher (*p* = 0.03) in the point-of-care TCD guided group (9/12, 75%) compared with the no point-of-care TCD guided group (2/9, 22.2%). The duration of hospital stay was significantly longer in the point-of-care TCD guided group compared with the no point-of-care TCD guided group (32.17 ± 19.82 vs. 11.67 ± 9.12 days, *p* = 0.006). Of the 11 survivors, 6 (54.5%) had PCPC scores of 1 or 2 at 6 months follow-up.

The 6-month neurological outcomes were significantly better (PCPC ≤2) in the point-of-care TCD guided group compared with the no point-of-care TCD guided group; however, this difference was not statistically significant (5/9, 55.6% vs. 1/2, 50%, *p* = 1.000) (Table 1). Treatment details and outcomes of the 21 patients with asphyxial OHCA are listed in Additional Table 2.

### TCD Findings

The pattern of Doppler spectral waveform, PI and the mean blood velocity of bilateral middle cerebral artery in the point-of-care TCD guided group are shown in Additional Table 3. The TCD categories of the MCA at days 1 and 3 did not significantly influence the 1-month mortality or 6-month neurological outcomes. However, a category 1 in the MCA at day 5 led to a significantly better survival compared with category 3 (9 of 9 children in the 1-month survival group vs. 1 of 3 children in the 1-month mortality group; *p* = 0.045), but not in the 6-month neurological outcomes. The maximum category of TCD findings during the first 5 days was also not associated with the 1-month survival or 6-month neurological outcomes. TCD findings of the MCA during the different treatment phases, in the 12 patients receiving point-of-care TCD guided cerebral resuscitation protocol between 1-month survival and 6-month neurologic outcomes, are listed in Table 2.

**Table 2:** The parameters of the middle cerebral artery during different time points in 12 patients receiving point-of-care transcranial Doppler ultrasound guided cerebral resuscitation protocol.

**Characteristic**	**1 month outcome**			**6 month neurological outcome**		
	**Survival** **(***n*** = 9)**	**Death** **(***n*** = 3)**	***p*** **-value**	**Favorable outcome** **(***n*** = 5)**	**Unfavorable outcome** **(***n*** = 7)**	***p*** **value**
Doppler spectrum waveform			0.250			1.000
Continuous	9	2		5	5	
Discontinuous	0	1		0	1	
**TCD Finding**						
Day 1			0.360			0.237
Category 1	3	2		1	4	
Category 2	4	0		3	1	
Category 3	2	1		1	2	
Days 2–3			0.193			0.454
Category 1	4	1		3	2	
Category 2	5	1		2	4	
Category 3	0	1		0	1	
Days 4–5			0.045^*^			0.470
Category 1	9	1		5	5	
Category 2	0	0		0	0	
Category 3	0	2		0	2	
Maximum TCD finding during the first 5 days		0.211				0.539
Category 1	2	1		1	2	
Category 2	5	0		3	2	
Category 3	2	2		1	3	

*TCD, transcranial Doppler; MCA, middle cerebral artery; ROSC, Return of spontaneous circulation*.

* *p < 0.05: statistically significant*.

## Discussion

Sustained intracranial hypertension and inadequate cerebral reperfusion after resuscitation can lead to catastrophic irreversible neurological injury and death. Serial point-of-care TCD examinations allow clinicians to assess the effectiveness of cerebral perfusion after resuscitation (10–16). The prognostic value of TCD for children after resuscitation in the study hospital have been previously published (10). Based on this previous TCD report, a consensus management protocol was achieved in the pediatric intensive care units. After following this algorithm in the present study, point-of-care TCD guided cerebral resuscitation was associated with a significantly better 1-month survival rate compared with no point-of-care TCD guided group in pediatric patients with asphyxial OHCA (*p* = 0.03).

The first step in TCD examinations after resuscitation is to distinguish the pattern of Doppler spectral waveform (9–12). A discontinuous Doppler spectral waveform can be regarded as a lethal sign, which indicates cerebral circulatory arrest (22–24). In addition, the presence of PI >2.0 with a low mean blood velocity indicated severe inadequate reperfusion (ischaemia/hypoperfusion), related to increased ICP. In these situations, aggressive systemic haemodynamic support by volume loading and vasoactive agents (19, 21), combined with hyperosmolar therapy with serum sodium 155–160 mEq/L and serum osmolality 320–340 mOsm/kg H_2_O, should be utilized to improve the increased ICP and produce sufficient diastolic flow on TCD examination (19, 20, 25).

The second step of TCD examination after resuscitation is to distinguish the PI and mean flow velocity. The predominant TCD findings after resuscitation were low mean flow velocity. An effective cerebral perfusion after resuscitation has a notable influence on the final neurological prognosis (6–8). Recently, Lovett et al. reported using TCD to evaluate cerebral blood flow velocity in children with global hypoxic ischemic (HI) events (26). They found the patients with favorable neurologic outcomes had a flow velocity near normal, whereas those with unfavorable outcomes had more extreme flow velocity, defined as 2 standard deviations above normal cerebral blood flow velocity (26). In the current study, a different goal for hyperosmolar therapy and mean arterial blood pressure was used according to the TCD finding. A normal or high mean flow velocity (category 1) TCD finding on day 5 led to significantly better 1-month survival compared with a category 3 finding (*p* = 0.045).

As the intracranial cerebral pressure was not available, there is no consensus on an optimal mean arterial blood pressure in children. In this study, we used a point-of-care TCD guided cerebral resuscitation protocol, and found that there was a lower maximum vasoactive-inotropic score and a higher serum sodium level in the point-of-care TCD guided group compared with the no point-of-care TCD guided group, although this did not reach statistical significance. However, point-of-care TCD guided cerebral resuscitation to achieve adequate cerebral perfusion by systematic haemodynamic optimization, using volume loading and vasoactive agents combined with different goals of hyperosmolar therapy, may improve the outcomes of comatose survivors after resuscitation.

Furthermore, impaired cerebral autoregulation has been associated with worse outcomes. Lovett et al. also investigated cerebral autoregulation with transient hyperaemic response ratio (THRR) using TCD in children with global HI events and concluded that the children with favorable outcomes seemed to have more periods of intact cerebral autoregulation compared to those with unfavorable outcomes (26). Sundgreen et al. found mean arterial pressure should be maintained at a higher level to secure cerebral perfusion in abnormal cerebral autoregulation (8). These studies suggest that TCD with individualized goal-directed therapy can be used to manage blood pressure and maintain cerebral autoregulation. Future research should focus on the potential clinical applications of point-of-care TCD guided cerebral hemodynamic status and optimal mean arterial blood pressure.

There were some limitations to the present study. First, it was a retrospective study in a limited cohort of children after resuscitation. Therefore, it is difficult to make a strong conclusion about point of care TCD guided management algorithm on outcome. In term of this issue, it needs a prospective, randomized and large sample study. However, there have been few reports in pediatric literature regarding point-of-care TCD guided management after resuscitation, and the novelty of this study may outweigh the limitation of small numbers. Second, it is difficult to investigate every possible confounder separately, such as the use of vasopressors, the strategy of ventilator support, and sedation and anesthesia, with respect to the cerebral blood flow and outcomes of post-cardiac arrest care. Finally, the TCDs were performed by one operator and the treating clinicians were aware of the results. Therefore, there is a risk of favorable/unfavorable neurological outcomes being a self-fulfilling prophecy. Nonetheless, exploring the potential of point-of-care TCD in this population is an important avenue for further research.

## Conclusions

A stepwise management algorithm based on the pattern of Doppler spectral waveform, PI and mean flow velocity of MCA on TCD examinations could guide the cerebral hemodynamic status of cerebral resuscitation during post cardiac arrest care. Adjust neurocritical care by point-of-care TCD guided protocol, therefore, may improve the outcomes of pediatric asphyxial OHCA. A large randomized controlled trial of point-of-care TCD guided cerebral resuscitation should be considered for pediatric OHCA.

## Data Availability Statement

The original contributions presented in the study are included in the article/Supplementary Material, further inquiries can be directed to the corresponding author/s.

## Ethics Statement

The studies involving human participants were reviewed and approved by the Chang Gung Memorial Hospital Institutional Review Board (IRB numbers: 201700975B0, 201700976B0, 201700977B0, and 201900302B0). Written informed consent for participation was not provided by the participants' legal guardians/next of kin because: Retrospective study.

## Author Contributions

J-JL conceived the study and drafted the manuscript. J-JL, H-CK, Y-JL, M-HH, M-CC, O-WC, and E-PL participated in data collection. S-HH, H-SW, and K-LL participated in the study's design and coordination. K-LL critically revised the manuscript for important intellectual content. All authors contributed to the article and approved the submitted version.

## Funding

This study was supported in part by grants from Chang Gung Memorial Hospital (CMRPG3G1311-2).

## Conflict of Interest

The authors declare that the research was conducted in the absence of any commercial or financial relationships that could be construed as a potential conflict of interest.

## Publisher's Note

All claims expressed in this article are solely those of the authors and do not necessarily represent those of their affiliated organizations, or those of the publisher, the editors and the reviewers. Any product that may be evaluated in this article, or claim that may be made by its manufacturer, is not guaranteed or endorsed by the publisher.

## Abbreviations

PCPC: Pediatric Cerebral Performance Category
ROSC: recovery of spontaneous circulation.
