# Supplementary material for: The Utility of a Point-of-Care Transcranial Doppler Ultrasound Management Algorithm on Outcomes in Pediatric Asphyxial Out-of-Hospital Cardiac Arrest – An Exploratory Investigation
Source: Front Med (Lausanne). 2022 Jan 28;8:690405. doi: 10.3389/fmed.2021.690405 (PMC8832099; doi:10.3389/fmed.2021.690405)
Supplement: Supplementary file 1 [file Table_1.docx]

**Additional table 1**. Clinical data and characteristics during resuscitation of the 21 children with asphyxial out-of-hospital cardiac arrest receiving therapeutic hypothermia

|  | Age (y) | Sex | Underlying disease | Interval of CPR to ROSC (min) | | | Serum pH | Initial glucose (mg/dL) | Initial lactate (mmol/L) | Post-cardiac arrest GCS | | PRISM III | PELODS |
| --- | --- | --- | --- | --- | --- | --- | --- | --- | --- | --- | --- | --- | --- |
| **Point-of-care TCD guided group (n=12)** | | | | | | | | |  | | | | |
| 1 | 0.5 | M | No | | 10 | 7.33 | | 266 | 71.7 | 3 | 26 | | 41 |
| 2 | 0.8 | M | No | | 30 | 6.88 | | 333 | 88.7 | 6 | 36 | | 32 |
| 3 | 0.6 | M | Achondroplasia | | 30 | 7.21 | | 301 | 72.5 | 3 | 27 | | 31 |
| 4 | 0.2 | M | No | | 10 | 7.11 | | 253 | 95.3 | 3 | 37 | | 31 |
| 5 | 0.3 | F | No | | 30 | 7.06 | | 153 | 77.0 | 6 | 27 | | 23 |
| 6 | 1.2 | M | Prematurity | | 9 | 6.99 | | 19 | 87.6 | 3 | 43 | | 52 |
| 7 | 0.2 | M | No | | 30 | 7.02 | | 363 | 84.4 | 3 | 42 | | 42 |
| 8 | 5.9 | M | No | | 15 | 7.20 | | 182 | 41.2 | 3 | 36 | | 41 |
| 9 | 0.4 | M | Developmental delay | | 23 | 7.13 | | 444 | 76.7 | 3 | 41 | | 31 |
| 10 | 4.0 | F | No | | 20 | 7.29 | | 249 | 26.9 | 3 | 39 | | 41 |
| 11 | 0.5 | M | No | | 40 | 6.75 | | 82 | - | 3 | 40 | | 52 |
| 12 | 0.9 | F | Respiratory | | 81 | 6.60 | | 466 | 211.8 | 3 | 51 | | 42 |
| **Non-point-of-care TCD guided group (n=9)** | | | | | | | | | | | | | |
| 13 | 9.9 | M | No | | 10 | 7.23 | | 87 | 53.4 | 3 | 40 | | 52 |
| 14 | 10.3 | M | No | | 20 | 7.33 | | 83 | 12.3 | 3 | 42 | | 42 |
| 15 | 0.4 | M | Prematurity | | 11 | 6.95 | | 321 | 131.6 | 3 | 41 | | 42 |
| 16 | 0.2 | F | Prematurity | | 8 | 7.14 | | 470 | 113.4 | 3 | 48 | | 51 |
| 17 | 0.3 | M | No | | 30 | 6.87 | | 167 | 155.6 | 3 | 40 | | 42 |
| 18 | 0.2 | M | No | | 10 | 7.18 | | 261 | 53.8 | 3 | 45 | | 41 |
| 19 | 0.1 | M | Neurologic | | 15 | - | | - | - | 3 | 40 | | 41 |
| 20 | 11.0 | M | No | | 23 | 6.93 | | 287 | 33.3 | 3 | 39 | | 41 |
| 21 | 11.3 | F | No | | 30 | 7.09 | | - | 107.1 | 3 | 46 | | 42 |

y: year; F: female, M: male; CP: cerebral palsy; CPR: cardiopulmonary resuscitation; ROSC: return of spontaneous circulation; GCS: Glasgow Coma Scale; PRISM: paediatric risk of mortality; PELODS: paediatric logistic organ dysfunction scores.
