# Supplementary material for: The Utility of a Point-of-Care Transcranial Doppler Ultrasound Management Algorithm on Outcomes in Pediatric Asphyxial Out-of-Hospital Cardiac Arrest – An Exploratory Investigation
Source: Front Med (Lausanne). 2022 Jan 28;8:690405. doi: 10.3389/fmed.2021.690405 (PMC8832099; doi:10.3389/fmed.2021.690405)
Supplement: Supplementary file 2 [file Table_2.docx]

**Additional table 2**. Treatment and outcomes of the 21 children with asphyxial out-of-hospital cardiac arrest receiving therapeutic hypothermia.

|  | Treatment | | | | | | Outcome | | | |
| --- | --- | --- | --- | --- | --- | --- | --- | --- | --- | --- |
|  | TTM | VIS | Ventilator duration (days) | | ICP control^#^ | | ICU stay (days) | Hospital length of stay (days) | 1-month survival | 6-month neurological outcomes |
|  |  |  |  |  | Serum sodium  (mEq/L)^#^ | Serum osmolality  (mosm/KgH_2_O) |  |  |  |  |
| **Point-of-care TCD guided group (n=12)** | | | | | |  |  | | | |
| 1 | HT | 15 | | 9 | 150 | 309 | 10 | 15 | Y | good |
| 2 | HT | 12.5 | | 17 | 146 | 293 | 26 | 47 | Y | good |
| 3 | HT | 16 | | 62 | 160 | 330 | 49 | 62 | Y | good |
| 4 | HT | 12.5 | | 9 | 161 | 333 | 14 | 22 | Y | good |
| 5 | HT | 17.5 | | 5 | 156 | 318 | 11 | 16 | Y | good |
| 6 | HT | 25 | | 24 | 152 | 320 | 37 | 59 | Y | poor |
| 7 | HT | 15 | | 42 | 156 | 317 | 42 | 42 | Y | poor ^a^ |
| 8 | HT | 20 | | 7 | 151 | 312 | 15 | 41 | Y | poor |
| 9 | HT | 15 | | 43 | 163 | 344 | 43 | 43 | Y | poor |
| 10 | HT | 20 | | 30 | 157 | - | 30 | 30 | N | -^a^ |
| 11 | HT | 25 | | 6 | 164 | 337 | 6 | 6 | N | -^a^ |
| 12 | HT | 45 | | 3 | 168 | 354 | 3 | 3 | N | -^b^ |
| **Non-point-of-care TCD guided group (n=8)** | | | | | |  | | | | |
| 13 | HT | 47.5 | | 3 | 162 | 366 | 3 | 3 | N | - ^b^ |
| 14 | HT | 15 | | 17 | 156 | 330 | 17 | 17 | N | -^a^ |
| 15 | HT | 14.5 | | 2 | 164 | 331 | 2 | 2 | N | -^c^ |
| 16 | HT | 15.5 | | 30 | 156 | - | 30 | 30 | N | -^a^ |
| 17 | HT | 40 | | 2 | 144 | - | 2 | 2 | N | -^a^ |
| 18 | HT | 15 | | 7 | 149 | 300 | 16 | 16 | Y | good |
| 19 | HT | 25 | | 13 | 150 | - | 13 | 13 | Y | poor |
| 20 | HT | 15 | | 14 | 151 | 304 | 14 | 14 | N | -^a^ |
| 21 | HT | 45 | | 8 | 150 | - | 8 | 8 | N | -^a^ |

TTM: targeted temperature management; HT: hypothermia therapy; NT: normothermia therapy; VIS: [Vasoactive-Inotropic Score](https://www.ncbi.nlm.nih.gov/pmc/articles/PMC4159673/); ICP: intracranial pressure;

^#^ The maximum serum level of sodium and osmolality during the first 3 days

^a^ Brain death or withdrawal for poor neurologic prognosis; ^b^ Cardiovascular failure / futility; ^c^ Respiratory failure / futility
