# Supplementary material for: The Utility of a Point-of-Care Transcranial Doppler Ultrasound Management Algorithm on Outcomes in Pediatric Asphyxial Out-of-Hospital Cardiac Arrest – An Exploratory Investigation
Source: Front Med (Lausanne). 2022 Jan 28;8:690405. doi: 10.3389/fmed.2021.690405 (PMC8832099; doi:10.3389/fmed.2021.690405)
Supplement: Supplementary file 3 [file Table_3.docx]

**Additional table 3.** Pattern of Doppler spectral waveform, pulsatility index and mean blood velocity of bilateral middle cerebral artery in transcranial Doppler ultrasound guided patients.

|  |  | Day 1 | | |  | Days 2-3 | | |  | Days 4-5 | | | | | |
| --- | --- | --- | --- | --- | --- | --- | --- | --- | --- | --- | --- | --- | --- | --- | --- |
|  |  | Waveform | PI | MV |  | Waveform | PI | MV |  | Waveform | | PI | | MV | |
| **Survival more than 1month** | | | | | | | | | | | | | | |  |
| **Favourable outcome (PCPC ≤ 2)** | | | | | | | | | | | | | | |  |
| 1 | R | Continuous | 1.10 | 52 | Continuous | | 0.95 | 87 | Continuous | | 1.04 | | 95 | |  |
|  | L | Continuous | 1.06 | 64 | Continuous | | 0.86 | 129 | Continuous | | 1.04 | | 68 | |  |
| 2 | R | Continuous | 0.92 | 74 | Continuous | | 0.80 | 133 | Continuous | | 0.77 | | 105 | |  |
|  | L | Continuous | 1.19 | 77 | Continuous | | 0.86 | 115 | Continuous | | 0.70 | | 120 | |  |
| 3 | R | Continuous | 2.07 | 34 | Continuous | | 0.56 | 150 | Continuous | | 0.56 | | 103 | |  |
|  | L | Continuous | 2.56 | 34 | Continuous | | 0.55 | 202 | Continuous | | 0.59 | | 87 | |  |
| 4 | R | Continuous | 1.52 | 36 | Continuous | | 1.37 | 54 | Continuous | | 0.62 | | 67 | |  |
|  | L | Continuous | 1.17 | 65 | Continuous | | 1.01 | 41 | Continuous | | 0.76 | | 91 | |  |
| 5 | R | Continuous | 1.00 | 107 | Continuous | | 1.50 | 119 | Continuous | | 1.10 | | 59 | |  |
|  | L | Continuous | 1.20 | 96 | Continuous | | 1.16 | 111 | Continuous | | 1.08 | | 84 | |  |
| **Unfavourable outcome (PCPC ≥ 3)** | | | | | | | | | | | | | | |  |
| 6 | R | Continuous | 0.82 | 58 | Continuous | | 1.06 | 69 | Continuous | | 0.99 | | 134 | |  |
|  | L | Continuous | 0.79 | 30 | Continuous | | 1.44 | 63 | Continuous | | 0.88 | | 73 | |  |
| 7 | R | Continuous | 1.15 | 84 | Continuous | | 1.63 | 52 | Continuous | | 0.85 | | 132 | |  |
|  | L | Continuous | 0.93 | 64 | Continuous | | 1.80 | 62 | Continuous | | 0.64 | | 73 | |  |
| 8 | R | Continuous | 0.82 | 44 | Continuous | | 0.98 | 62 | Continuous | | 0.78 | | 85 | |  |
|  | L | - | - | - | Continuous | | 1.06 | 46 | Continuous | | 0.66 | | 98 | |  |
| 9 | R | Continuous | 2.83 | 25 | Continuous | | 1.53 | 31 | Continuous | | 0.94 | | 82 | |  |
|  | L | Continuous | 2.74 | 15 | Continuous | | 1.15 | 24 | Continuous | | 0.94 | | 60 | |  |
| **Mortality within 1 month** | | | | | | | | | | | | | | |  |
| **Unfavourable outcome (PCPC ≥ 3)** | | | | | | | | | | | | | | |  |
| 10 | R | Continuous | 0.69 | 62 | Continuous | | 0.53 | 97 | Continuous | | 0.75 | | 115 | |  |
|  | L | Continuous | 0.69 | 61 | Continuous | | 0.46 | 83 | Continuous | | 0.88 | | 99 | |  |
| 11 | R | Continuous | 2.53 | 33 | Continuous | | 1.67 | 28 | Continuous | | 2.29 | | 21 | |  |
|  | L | Continuous | 2.26 | 21 | Continuous | | 1.38 | 28 | Continuous | | 2.12 | | 21 | |  |
| 12 | R | Continuous | 1.05 | 63 | Discontinuous | | - | - | - | | - | | - | |  |
|  | L | Continuous | 0.96 | 64 | Discontinuous | | - | - | - | | - | | - | |  |

PI: pulsatility index; MV: mean blood velocity; R: right; L: left; PCPC: paediatric cerebral performance category.
